# Diagnostic and prognostic value of *STAP1* and *AHNAK* methylation in peripheral blood immune cells for HBV-related hepatopathy

**DOI:** 10.3389/fimmu.2022.1091103

**Published:** 2023-01-13

**Authors:** Libo Sun, Junfeng Lu, Kang Li, Haitao Zhang, Xiaofei Zhao, Guangming Li, Ning Li

**Affiliations:** ^1^ General Surgery Center, Beijing YouAn Hospital, Capital Medical University, Beijing, China; ^2^ Department of Liver Disease Center, Beijing YouAn Hospital, Capital Medical University, Beijing, China; ^3^ Biomedical Information Center, Beijing YouAn Hospital, Capital Medical University, Beijing, China

**Keywords:** STAP1, AHNAK, DNA methylation, hepatopathy, diagnosis, prognosis, HBV

## Abstract

**Introduction:**

Although we had identified that the methylation of AHNAK was a good diagnostic marker for hepatopathy, here we speculate that there was also another marker, STAP1, whose methylation also involved in the detection of hepatopathy.

**Methods:**

We investigated the methylation levels of the AHNAK and STAP1 in peripheral blood mononuclear cells of chronic hepatitis B (CHB) patients, compensatory liver cirrhosis (CLC) patients, decompensated liver cirrhosis (DCLC) patients, hepatocellular carcinoma (HCC) patients and healthy controls by methylation-specific PCR. We also evaluated the differences and changes of methylation and expression of AHNAK and STAP1 at different stages of liver disease using the TCGA and GEO public datasets.

**Results:**

Methylation level of STAP1 in PBMC was positively correlated with the course of liver cancer. The combination of AHNAK and STAP1 methylation was able to predict differrent HBV related hepatopathy. The GEO datasets also supported that the methylation of AHNAK and STAP1 was associated with different types of hepatopathy. The TCGA data showed that the levels of methylation and expression of STAP1 were down-regulated in HCC. We also found the STAP1 methylation level in PBMC and T cells was associated with age, gender, alcohol drinking and anti-HBe. Hyper-methylation of STAP1 was correlated with the poor prognosis of patients but its expression had no association.

**Conclusion:**

We concluded that combination of AHNAK and STAP1 methylation in peripheral blood immune cells can be used as a diagnostic marker for HBV related hepatopathy and STAP1 methylation may be a potential prognostic marker for HBV related HCC. Our clinical study registration number was ChiCTR2000039860.

## Introduction

Hepatocellular carcinoma (HCC) is the fifth most common neoplasm and the third most common cause of cancer-related deaths in the world (1–3). Almost all patients with liver cirrhosis (4) and a large portion of those with chronic liver disease eventually progress to liver fibrosis and HCC (5). Because the majority of people with liver cirrhosis are asymptomatic and because HCC patients frequently come with hepatitis and HCC symptoms at an advanced stage, the effectiveness of treatment is frequently limited (6, 7). Therefore, finding efficient biomarkers for early diagnosis detection, therapy evaluation, and prognosis prediction of liver cancer is therefore very valuable.

Emerging evidence suggests that many genetic and epigenetic abnormalities are involved in the development of HCC (8). DNA methylation is recognized as one of the first epigenetic modifications discovered, which can regulate gene expression by influencing the chromatin structure, DNA conformation, DNA stability, and DNA–protein action mode (9). A growing number of studies have shown that DNA methylation can be used as a diagnostic and prognostic molecular marker for multiple diseases (10–12). Our previous study has reported that the promoter methylation level of *AHNAK* gene in peripheral blood mononuclear cells (PBMC) was associated with the increase of the severity of liver diseases, and *AHNAK* can be employed as an early diagnostic marker for HCC (13). However, a few patients did not conform to the *AHNAK*-based rule. In addition, our previous research also showed that *STAP1* methylation in PBMC is also associated with the progression of hepatitis B virus (HBV)-related liver diseases. Therefore, in this study, we collected new patient samples to investigate the correlation between the methylation of another gene, *STAP1*, as well as *AHNAK*, and the progression of hepatitis and HCC and to assess the diagnostic effect of the combination using the genes *STAP1* and *AHNAK*.

The protein STAP1 is a substrate of tyrosine-protein kinase Tec, participating in a positive feedback loop by upregulating the activity of tyrosine-protein kinase Tec. Although STAP1 is reported to be involved in B cell receptor signaling (14) and in the prognosis gene signature of lung adenocarcinoma (15), the role of STAP1 in HCC is not clear. We here used methylation-specific polymerase chain reaction (MSP) to study the *STAP1* and *AHNAK* methylation level in hepatitis B patients and its correlation to the progress of HCC from BCLC stage 0 to stage C. We also explored the association of methylation level and expression level of *STAP1* and *AHNAK* to the different stages of HCC and survival of patients using several public datasets. The current study aimed to explore the diagnostic and prognostic value of *STAP1* methylation in liver cirrhosis and HCC combined with *AHNAK*.

## Materials and methods

### Patients and collection of samples

A total of 302 HBV-infected patients in Beijing YouAn Hospital, Capital Medical University were recruited during June 2014 to August 2020, including 46 cases of chronic hepatitis B (CHB), 46 cases of compensatory liver cirrhosis (CLC), 53 cases of decompensated liver cirrhosis (DCLC), 157 cases of HCC (stage 0 = 34, stage A = 42, stage B =19, and stage C = 62). In total, 22 healthy volunteers were recruited as controls during a routine physical examination, and none of them had hepatobiliary diseases before the peripheral blood sample collection. The whole PBMC suspension was prepared by taking 3–5 ml peripheral blood of the patients into EDTA anticoagulant tubes. T cells were also isolated from the PBMC by using the magnetic activated cell sorter (MACS^®^) T-cell isolation kit following the manufacturer’s instructions. All the participants signed informed consents, and the study was approved by the medical ethics committee of Beijing YouAn Hospital (BJYACE-201721).

### DNA extraction, bisulfite modification, and MSP

**Figure 1:**
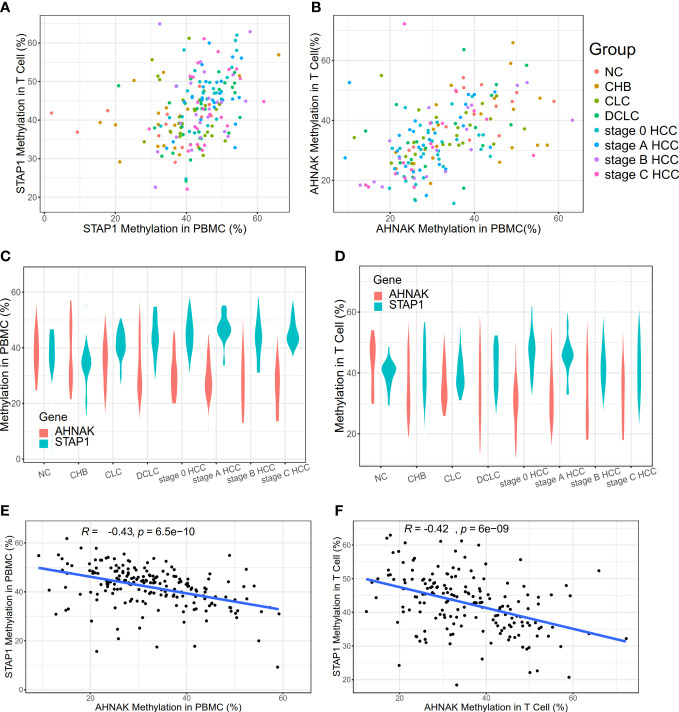
Methylation level of *AHNAK* and *STAP1* in peripheral blood mononuclear cells (PBMC) and T cells in different liver diseases. **(A)** Methylation levels of *STAP1* in PBMC and T cells. **(B)** Methylation levels of *AHNAK* in PBMC and T cells. **(C)** Methylation levels of *AHNAK* and *STAP1* in PBMC at different disease stages. **(D)** Methylation level of *AHNAK* and *STAP1* in T cells at different disease stages. **(E, F)** Negative correlation of methylation of *AHNAK* and *STAP1* in PBMC and T cells.

Genomic DNA was extracted from PBMC or T cells of patients and healthy controls using the QIAamp DNA mini kit (Qiagen NV, Venlo, The Netherlands) in line with the manufacturer’s protocol. The quality and the quantity of the isolated DNA were measured by NanoDrop 2000 Spectrophotometer (Thermo Fisher Scientific). The bisulfite treatment of genomic DNA was processed using an EpiTect Fast DNA Bisulfite Kit (Qiagen NV). The bisulfite-treated DNA samples were next kept at -20°C for further use. After analyzing a 2,000-bp region upstream of the transcription start site (*AHNAK* or *STAP1* promoter region), one CpG island was identified (**Figure 1A**). With bisulfite-treated DNA as the template, the methylation pattern in the CpG island within *AHNAK* or *STAP1* promoter was measured by MSP, and the MSP primers are presented in **Supplementary Table S1**. The MSP primers were designed according to the principle described previously (16). The reaction condition for MSP was 10 min at 95°C, followed by 40 cycles of 30 s at 94°C and 30 s at 72°C, and then 5 min of extension at 72°C. After having been separated on 1.5% agarose gels, the MSP products were then stained by ethidium bromide and visualized under a UV spectrophotometer. Water blanks served as the negative control. We obtained the methylation levels of *AHNAK* or *STAP1* in PBMC or T cells.

### Public datasets used

The expression and methylation data of HCC in the LIHC project in The Cancer Genome Atlas (TCGA) (17) was used to confirm the correlation of *AHNAK* and *STAP1* with the HCC and the overall survival of patients. DNA methylation data from the Gene Expression Omnibus (GEO) database were employed to investigate the methylation profile of *AHNAK* and *STAP1* in the different types of liver cirrhosis or HCC: (1) methylation profiling of 1,204 HCC patients, 392 patients with CHB or liver cirrhosis, and 958 healthy individuals and patients with benign liver lesions (GSE112679); (2) methylation profiling of 34 healthy liver tissues and 122 primary liver disease tissues arising in the setting of chronic HBV or C viral infection, alcoholism (EtOH) (GSE60753); and (3) methylation profiling of 48 HBsAg carriers who developed HCC and 48 HBsAg carriers without HCC during follow-up (GSE78904). The normalized data of those GEO datasets were downloaded through the GEOquery package in R.

### Statistical analysis

All 302 HBV-infected patients in Beijing YouAn Hospital were included in the statistical analysis. Student’s *t*-test and one-way analysis of variance (ANOVA) were used to compare the differences of *AHNAK* or *STAP1* methylation between groups. Spearman’s correlation was carried out to compare the difference of continuous variables. Linear regression was utilized to compare the difference of discrete variables. Correlations between the methylation levels of STAP1 or AHNAK and the clinical characteristics (including ages, sex, alcohol drinking, and anti-HBeAb) were determined *via* Spearman rank correlation analysis. Using the data of patients with information of overall survival (OS), we investigated the association of methylation or expression of *STAP1* as well as *AHNAK* to the patients’ OS. We grouped the patients into uppers and lowers according to the median of methylation or the expression level of *STAP1* or *AHNAK*. Kaplan–Meier curves with log-rank test were used for the overall survival. Statistical significance was defined as two-tailed *P <*0.05 for all analyses. To assess the diagnostic value of STAP1 and AHNAK methylation levels as markers for CHB, CLC, DCLC, or HCC, respectively, we performed receiver operating characteristic (ROC) analysis based on their methylation levels and used the area under the curve (AUC) as an assessment of diagnostic accuracy. The ROC curve analysis was performed using SPSS19.0 statistical software. Other data were analyzed using statistical software R3.1.1 or GraphPad Prism version 6.0.

## Results

### The methylation level of *STAP1* in PBMC was positively correlated with the course of liver cancer, while *AHNAK* had the reverse correlation

We found out that there was no significant difference in the methylation level of *STAP1* or *AHNAK* between the PBMC and T cells (**Figures 1A, B**). The *STAP1* methylation level was positively correlated with the severity of the liver disease not only in the PBMC but also the T cells, while *AHNAK* showed a negative correlation (**Figures 1C, D**). Thus, there was a reverse correlation between *AHNAK* and *STAP1* in the PBMC and T cells (**Figures 1E, F**). The ANOVA test suggested that the methylation levels of *AHNAK* and *STAP1* both showed significant differences among different groups whether in the PBMC or T cells (**Table 1**). A further two-paired comparison of *STAP1* methylation levels demonstrated that there existed significant differences between NC and DCLC and HCC (stage 0, A, B, C), between CHB and DCLC and HCC, and between CLC and DCLC, stage 0 HCC, and stage C HCC in the PBMC. There were significant differences between NC, CHB, CLC, and HCC stage 0 and HCC stage A in the T cells (**Supplementary Tables S2–S5**).

**Table 1:** *AHNAK* and *STAP1* methylation level among different groups in peripheral blood mononuclear cells (PBMC) and T cells.

Gene	Sample	df	Sum square	Mean square	*F*-value	Pr (>*F*)	Significance
*STAP1*	PBMC	7	3,527	503.8	7.928	1.34E-08	***
	T cell	7	1,141	163.04	2.387	0.0229	*
*AHNAK*	PBMC	7	2,932	418.9	4.251	0.000204	***
	T cell	7	2,005	286.4	2.37	0.0239	*

*P < 0.05, ***P < 0.001.

### Diagnostic value of *STAP1* and *AHNAK* methylation level in patients with different liver diseases

**Figure 2:**
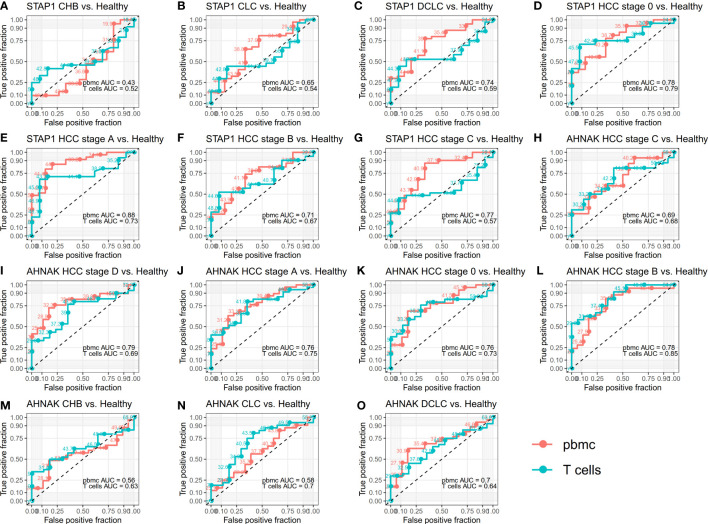
Receiver operating characteristic (ROC) curves for whether *STAP1* or *AHNAK* methylation in peripheral blood mononuclear cells (PBMC) can be used as an indicator for the diagnosis of liver disease. **(A–G)** ROC curves for methylation of *STAP1* in PBMC and T cells. **(H-O)**, ROC curves for methylation of *AHNAK* in PBMC and T cells.

To further verify whether *STAP1* or *AHNAK* methylation in PBMC can be used as an indicator for the diagnosis of liver diseases, the ROC curve was drawn for the analysis (**Figure 2**). The *STAP1* and *AHNAK* methylation levels were not highly specific for identifying CHB patients. *STAP1* methylation was also not specific for predicting patients with CLC (AUC = 0.65 in PBMC and AUC = 0.54 in T cells); however, *AHNAK* methylation in T cells was predictive of CLC (AUC = 0.7). The methylation of either *STAP1* or *AHNAK* in PBMC may be predictive of DCLC, with AUCs of 0.74 and 0.70, respectively. Either *STAP1* or *AHNAK* demonstrated pretty high specificity in predicting HCC, where the AUC of methylation of either *STAP1* or *AHNAK* in PBMC was mostly greater than 0.70.

### Methylation level of *AHNAK* and *STAP1* also showed significant differences among different types of liver diseases in the public datasets

**Figure 3:**
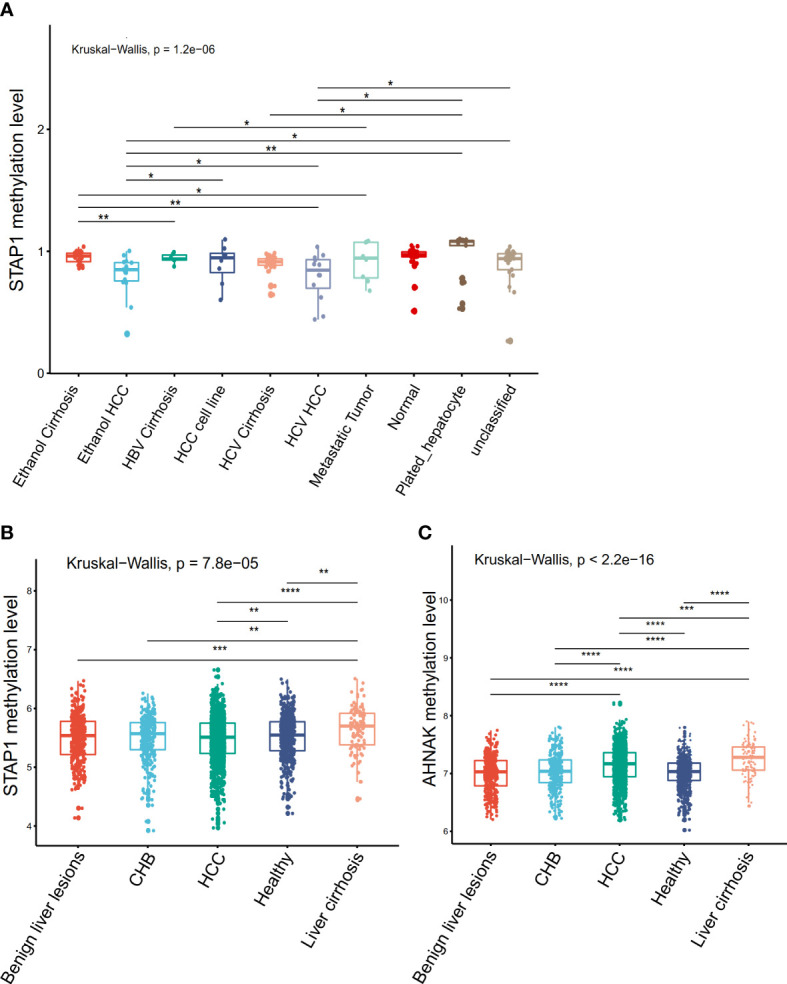
Comparison of the methylation of *AHNAK* and *STAP1* among different types of liver disease in the Gene Expression Omnibus datasets. **(A)** Comparisons in *STAP1* methylation among the different types of liver cirrhosis and hepatocellular carcinoma (HCC) patients (GSE60753 data). **(B)** Comparisons in *STAP1* methylation among the chronic hepatitis B (CHB), liver cirrhosis, and HCC patients and benign liver lesions (GSE112679 data). **(C)** Comparisons in *AHNAK* methylation among the CHB, liver cirrhosis, and HCC patients and benign liver lesions (GSE112679 data). *P < 0.05, **P < 0.01, ***p < = 0.001, ****p < = 0.0001.

In order to confirm the association of *STAP1* and *AHNAK* with liver diseases, we further analyzed the *AHNAK* and *STAP1* methylation levels in the different types of liver cirrhosis or HCC using the GEO datasets. In the GSE60753 data, there was a significant difference in *STAP1* methylation level among liver cirrhosis and HCC (Kruskal−Wallis test, *p* = 1.2e−06) (**Figure 3A**). In the GSE112679 data, the methylation level of *STAP1* was also significantly higher than in healthy controls and CHB and HCC patients (**Figure 3B**). In the GSE112679 data, *AHNAK* also showed a significantly high methylation level in liver cirrhosis than other types, and it was significantly higher in HCC compared with benign liver lesions and CHB (**Figure 3C**).

### Lower methylation level and expression level of *STAP1* and *AHNAK* in liver cancer tissues

**Figure 4:**
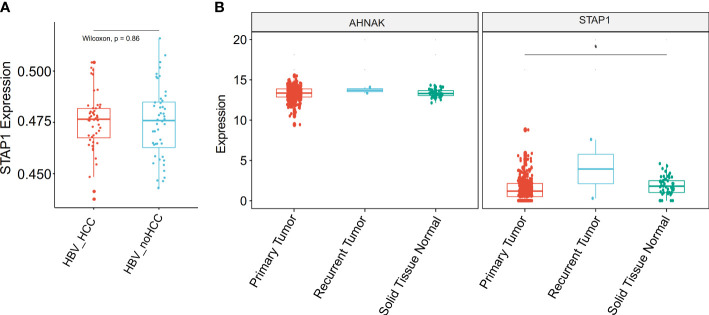
Comparison of the methylation and expression of *AHNAK* and *STAP1* between hepatocellular carcinoma (HCC) and normal patients. **(A)** Comparison of the methylation level of *STAP1* between HBsAg carriers who developed HCC and HBsAg carriers who did not. **(B)** Comparison of the expression level of *AHNAK* and *STAP1* between tumor and normal tissues in The Cancer Genome Atlas data. *P < 0.05.

We found that the cancer tissues displayed a lower methylation level of *STAP1* than the adjacent tissues (by *t*-test, *P* < 0.05) (**Table 2**). However, there was no significant difference between HBsAg carriers who developed HCC and HBsAg carriers who did not during follow-up in the GSE78904 data (**Figure 4A**).

**Table 2:** Methylation level of *STAP1* between cancer tissues and adjacent tissues.

CpG	Location	median_pt	median_pn	P_pt	FDR_pt
cg04398282	chr4:67558538-67558539	0.845	0.9005	1.70E-05	7.11E-05
cg09632271	chr4:67575504-67575505	0.6945	0.776	0.000357387	0.00110967
cg12879425	chr4:67558777-67558778	0.4535	0.6875	1.63E-06	8.68E-06

Pt, hepatocellular carcinoma tumor; Pn, normal tissue.

As gene methylation is able to regulate the gene expression of itself, we analyzed the expression levels of *STAP1* between primary tumor and solid normal tissue in TCGA data. We found that *STAP1* had a lower expression in primary tumor, while *AHNAK* showed no significant difference (Wilcoxon rank sum test, *P* < 0.05) (**Figure 4B**).

### *STAP1* methylation level was pertinent to age, sex, alcohol drinking, and anti-HBe

**Figure 5:**
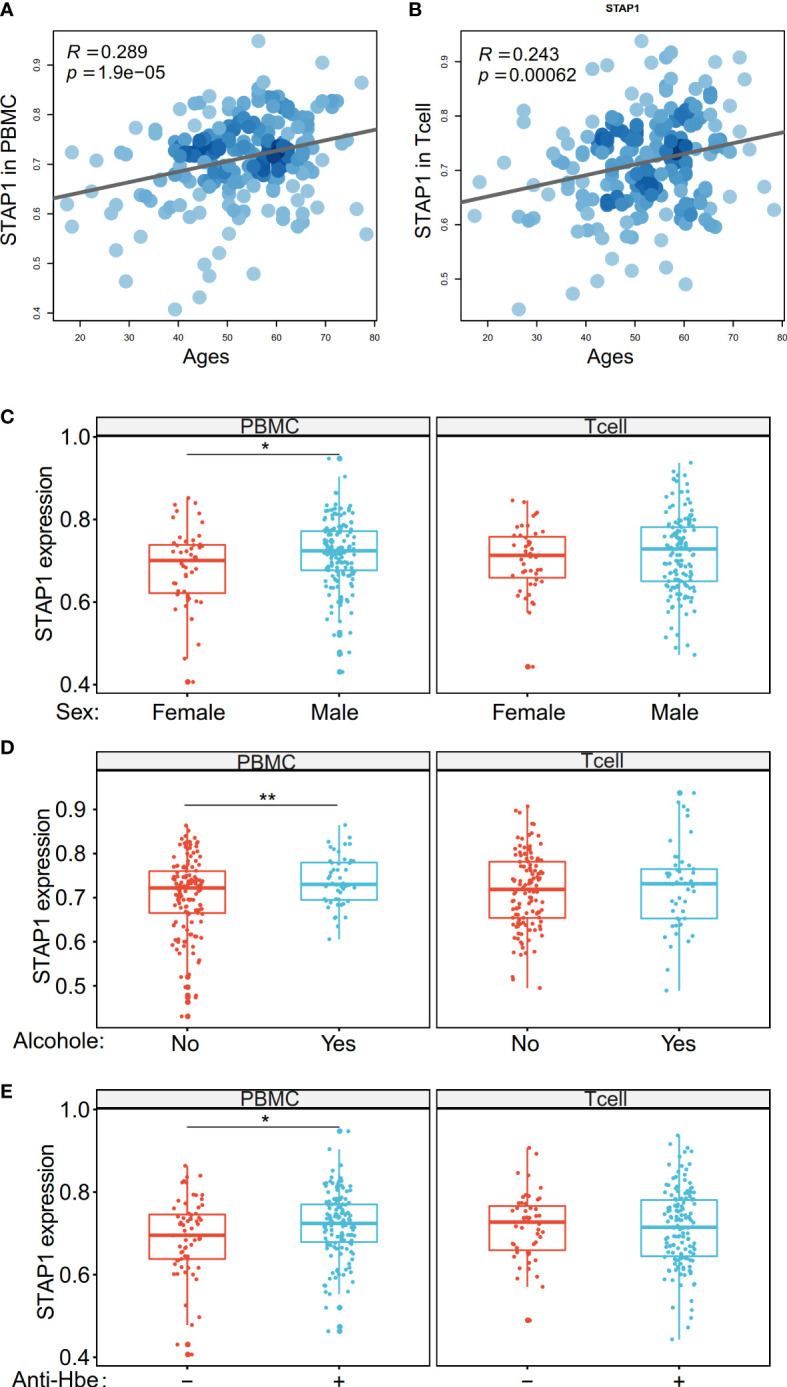
The methylation level of *STAP1* was correlated with age, sex, alcohol drinking, and anti-HBe in peripheral blood mononuclear cells (PBMC) and T cells. **(A, B)** Correlation of *STAP1* methylation level with ages in PBMC and T cells. **(C)** Difference of *STAP1* methylation in different genders in PBMC and T cells. **(D)** Difference of *STAP1* methylation between PBMC and T cells relative to alcohol abuse. **(E)** Difference of *STAP1* methylation between anti-HBe+ and anti-HBe- patients in PBMC and T cells. *P < 0.05, **P < 0.01.

In our new collection of patients with HBV, we found that the *STAP1* methylation level was positively correlated with age not only in the PBMC (*R* = 0.289, *p* = 1.9e-5) but also in T cells (*R* = 0.243, *p* = 6.2e-4) (**Figures 5A, B**). The *STAP1* methylation level in the PBMC was significantly higher in male than in female patients, while there was no significant difference in T cells (**Figure 5C**). Similarly, the *STAP1* methylation level in the PBMC was significantly higher in the alcohol-drinking patients than in the non-alcoholic patients and higher in the anti-HBe+ patients than in the anti-HBe- patients, but it showed no significant difference in T cells (**Figures 5D, E**). In the same set of patients, the *AHNAK* methylation level, as it was negatively correlated with *STAP1* methylation, was lower in the older patients and was significantly higher in the female patients, in the non-alcoholic patients, and in non-smoking patients (**Supplementary Figure S1**).

**Figure 6:**
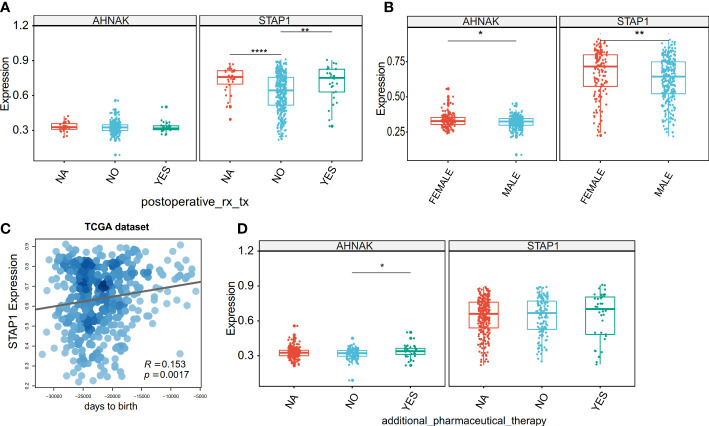
Association between the methylation level of *STAP1* or *AHNAK* and clinical characteristics in The Cancer Genome Atlas data. **(A)** Difference of *STAP1* and *AHNAK* methylation between whether postoperative rx tx. **(B)** Difference of *STAP1* and *AHNAK* methylation between different genders. **(C)** Correlation of *STAP1* methylation with ages. **(D)** Difference of *STAP1* and *AHNAK* methylation between whether additional pharmaceutical therapy was used or not. *P < 0.05, **P < 0.01, ****P < 0.0001.

On the other hand, we verified the relationship between the clinical characteristics and *STAP1* and *AHNAK* in the HCC patients using the TCGA data. The *STAP1* methylation level was associated with postoperative rx tx (**Figure 6A**), gender (**Figure 6B**), and age (**Figure 6C**), while the *AHNAK* methylation level was associated with additional pharmaceutical therapy (**Figure 6D**) and sex (**Figure 6B**).

### The combination of *AHNAK* and *STAP1* methylation levels was associated with the overall survival of patients

**Figure 7:**
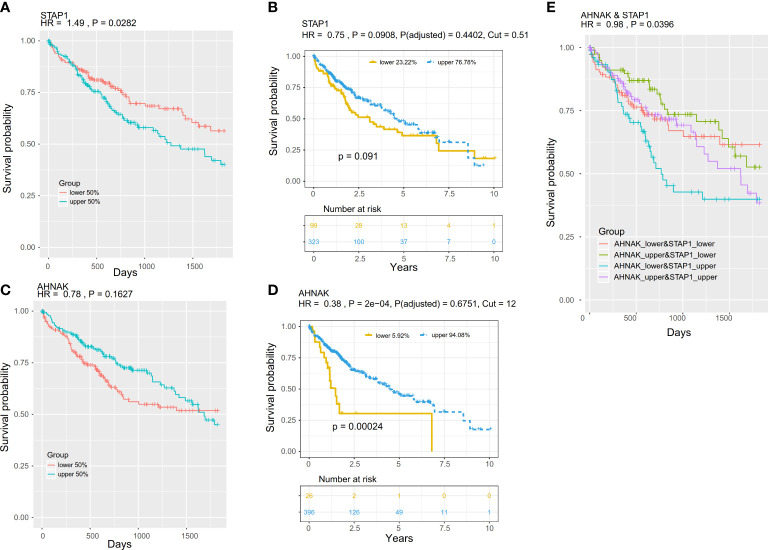
Prognostic value of the methylation and expression of *AHNAK* and *STAP1*. The K–M survival curves show the overall survival of patients in The Cancer Genome Atlas project. The patients were stratified (upper and lower) according to the median of the methylation or expression level. **(A, B)** K–M survival curves for *STAP1* methylation **(A)** and expression **(B)**. **(C, D)** K–M survival curves for the *AHNAK* methylation **(C)** and expression **(D)**. K–M survival curves for the combination of methylation of *AHNAK* and *STAP1*. **(E)** OS of different AHNAK &STAP1 combined groups.

The result suggested that methylation of *STAP1* was significantly associated with OS, and it was a risk factor (HR = 1.49, *P* = 0.0282), but the expression level of STAP1 was not significantly associated with OS (HR = 0.75, *P* = 0.0908) (**Figures 7A, B**). Neither the methylation nor the expression of *AHNAK* was significantly associated with OS (HR <1, *P* > 0.05) (**Figures 7C, D**). However, there was a significant distinction in OS (HR = 0.98, *P* = 0.0396) when using the combination of methylation of *STAP1* and *AHNAK* with the patients who carried lower *AHNAK* and upper *STAP1* having poor OS (**Figure 7E**).

## Discussion

*AHNAK*, as a good diagnostic marker [with AUC as 0.883 (*P* < 0.001) in the diagnosis of CHB, 0.885 (*P* < 0.001) in the diagnosis of compensatory liver cirrhosis, 0.955 (*P* < 0.001) in the diagnosis of decompensated liver cirrhosis, and 0.981 (*P* < 0.001) in the diagnosis of hepatocellular carcinoma], has been identified in our previous research (13). We here employed a new set of patients to investigate the methylation level of a novel marker, *STAP1*, as well as *AHNAK*, in the PBMC and T cells also in different course samples of hepatitis B. We obtained a similar result for *AHNAK*. In this study, we investigated the methylation of *STAP1* and found that the methylation of *STAP1* was negatively correlated with the methylation of *AHNAK*. Although *AHNAK* can distinguish hepatitis B patients to a certain extent in the methylation, the combination of these two markers can achieve better performance. It is reported that *STAP1* was associated with hypercholesterolemia (18–20). However, there were a few reports about *STAP1* in cancer (14, 15) and no publication for hepatopathy. Therefore, our study is the first to discover the relationship of methylation of *STAP1* with hepatopathy and HCC. The combination of methylation of *STAP1* and *AHNAK* indeed achieved a better performance in distinguishing the different types of hepatopathy patients, which suggests the diagnostic value of *STAP1*.

On the other hand, using the expression data and the methylation data of public datasets, we investigate the relationship between the methylation and the expression of *STAP1* and *AHNAK*. For *AHNAK*, there was hypo-methylation shown in the HCC than in adjacent tissues in TCGA dataset but conversely in the GEO data. In our data, we found a negative correlation of the methylation of *AHNAK* and *STAP1* in the PBMC. However, in the public data, there was hyper-methylation of both *AHNAK* and *STAP1* in liver cirrhosis and hypo-methylation both in the HCC and adjacent tissues. The expression of the gene can be downregulated by the hyper-methylation of its promoter region through inhibition of the binding of transcription factors or recruitment of methyl-CpG-binding proteins to silence the gene (21, 22). However, with the data including expression and methylation simultaneously in TCGA, we found decreases in both the methylation and the expression levels of *STAP1* in HCC. We think that the contradiction mentioned above is due to the difference between tissue and blood or the difference between the detected positions of methylation.

Similar to *AHNAK*, *STAP1* methylation was also related to alcohol drinking, which suggests that these bad habits do affect the occurrence of cancer. Liver cancer is often diagnosed at an advanced stage and has a high mortality rate (23, 24). Therefore, early diagnosis is very important. The combination of these two genes can be used in the early screening and prognostic judgments and provide a basis for the formulation of treatment strategies.

To date, little is known about the role of STAP1 in hepatocarcinogenesis. There are several intracellular signaling pathways that derive from the STAP-1 and STAP-2 members of the signal-transducing adaptor protein (STAP) family. These proteins have Pleckstrin homology in their N-terminal sections and SRC homology 2 domains in their middle regions, which are common architectures for adaptor proteins. During carcinogenesis and inflammatory/immune reactions, STAP proteins interact with the inhibitor of nf-κb kinase complex and activator of transcription 3. In hepatocellular carcinoma, the aberrant methylation of STAP1 may also contribute to hepatocarcinogenesis *via* these signaling pathways. However, there are some limitations in the present study. First, only PBMC and T cell specimens were used in this study, although the methylation and the expression status in liver tissues were investigated using the public data. The finding in the PBMC and T cell for those two genes was not overall consistent with that in the tissues, and even the different datasets showed different status. Thereby, the intrahepatic methylation of *AHNAK* and *STAP1* still needs to be studied in the future. However, the main purpose of our study was to identify the potential clinical diagnostic marker for HBV-related patients, and it is beneficial to use peripheral blood immune cells in the clinical application. In future research, we should investigate the methylation status of *STAP1* in different tissues. Second, the MSP approach that we used here only tests whether methylation occurs; other approaches such as gene sequencing would be more helpful. However, it is undeniable that MSP is of excellent specificity, sensitivity, and operability in frequent detection. Finally, the mechanisms underlying *STAP1* and *AHNAK* have not been studied here. Especially for *STAP1*, little is known about its function, and thus in the future, an experimental investigation about it should be carried out to understand its functional role in HBV-related hepatopathy.

## Data availability statement

The datasets presented in this study can be found in online repositories. The names of the repository/repositories and accession number(s) can be found in the article/**Supplementary Material**.

## Ethics statement

The studies involving human participants were reviewed and approved by the medical ethics committee of Beijing YouAn Hospital (BJYACE-201721). The patients/participants provided their written informed consent to participate in this study. Written informed consent was obtained from the individual(s) for the publication of any potentially identifiable images or data included in this article.

## Author contributions

LS contributed to sample and data acquisition and manuscript drafting. JL, KL, HZ, and XZ provided sample and data acquisition and technical support. GL and NL made substantial contributions to the conception and design, funding, and supervision of the study. All authors contributed to the article and approved the submitted version.
