# Supplementary material for: Diagnostic and prognostic value of STAP1 and AHNAK methylation in peripheral blood immune cells for HBV-related hepatopathy
Source: Front Immunol. 2023 Jan 13;13:1091103. doi: 10.3389/fimmu.2022.1091103 (PMC9880311; doi:10.3389/fimmu.2022.1091103)
Supplement: Supplementary file 1 [file DataSheet_1.docx]

Supplementary Material

# Supplementary Tables

**Supplementary Table 1.** Amplification and sequencing primers of target genes.

**Supplementary Table 2.** Comparison of *AHNAK* methylation levels in PBMC between different groups.

**Supplementary Table 3.** Comparison of *AHNAK* methylation levels in T cells between different groups.

**Supplementary Table 4.** Comparison of *STAP1* methylation levels in PBMC between different groups.

**Supplementary Table 5.** Comparison of *STAP1* methylation levels in T cells between different groups.

**Supplementary Figures**

**Supplementary Figure 1.** 1A, The correlation of *AHNAK* methylation in PBMC with age. 1B, The correlation of *AHNAK* methylation in T cells with age. 1C, Differences of *AHNAK* methylation in PBMC and T cells between male and female group. 1D, Differences of *AHNAK* methylation in PBMC and T cells between drinking and no drinking group. 1E, Differences of *AHNAK* methylation in PBMC and T cells between smoking and no smoking group.
