# Supplementary figures and images for: Diagnostic and prognostic value of STAP1 and AHNAK methylation in peripheral blood immune cells for HBV-related hepatopathy
Source: Front Immunol. 2023 Jan 13;13:1091103. doi: 10.3389/fimmu.2022.1091103 (PMC9880311; doi:10.3389/fimmu.2022.1091103)

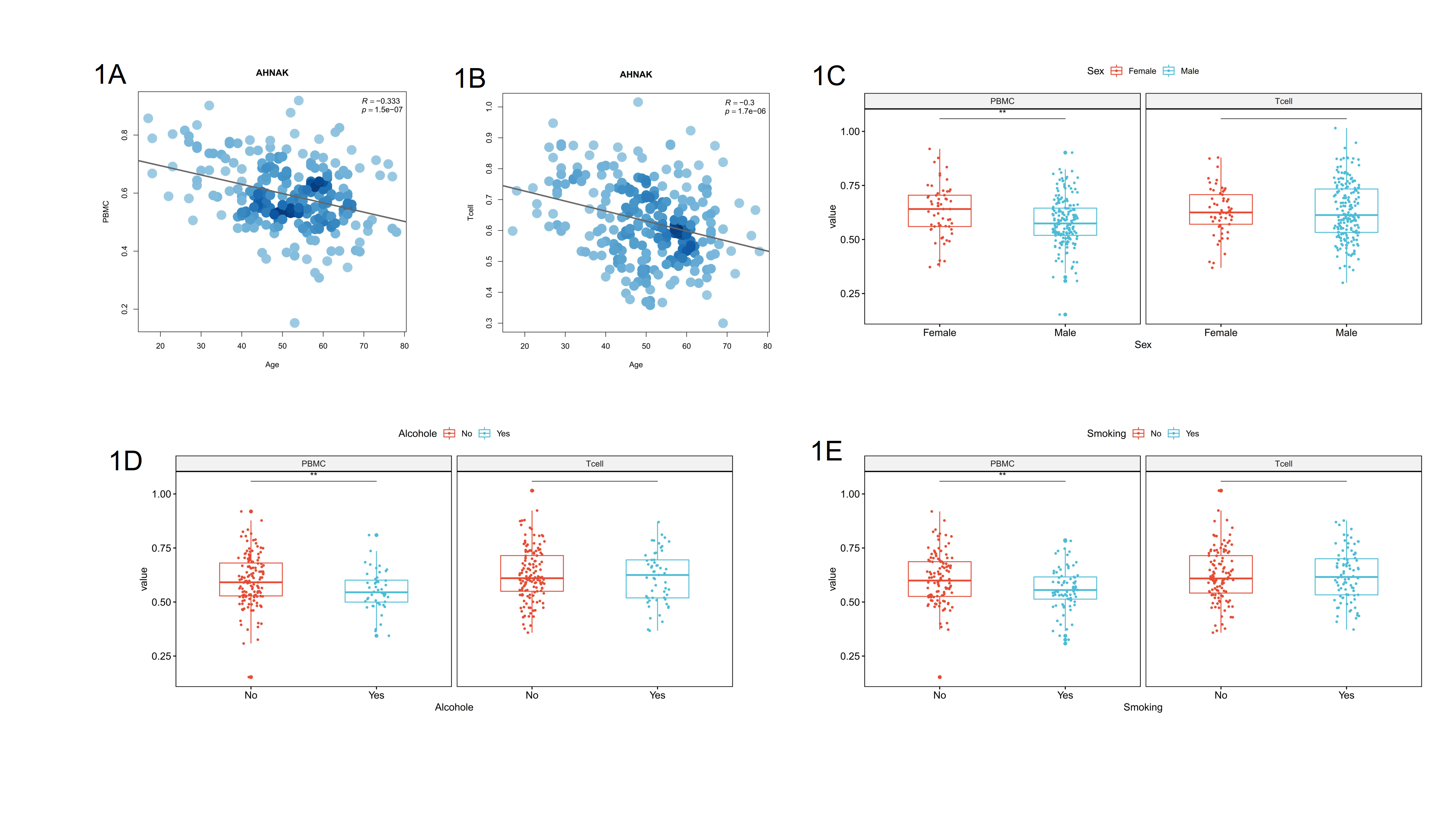

Supplement: Supplementary file 2 [file Image_1.tif]
