# Supplementary material for: Diagnostic and prognostic value of STAP1 and AHNAK methylation in peripheral blood immune cells for HBV-related hepatopathy
Source: Front Immunol. 2023 Jan 13;13:1091103. doi: 10.3389/fimmu.2022.1091103 (PMC9880311; doi:10.3389/fimmu.2022.1091103)
Supplement: Supplementary file 3 [file Table_1.docx]

**Supplemental Table 1**, Amplification and sequencing primers of target genes

| Gene | Primer | Sequence(5' -3') | Size(bp) |
| --- | --- | --- | --- |
| *AHNAK* | out foward | GGATGTGTCGAGTAGTAGGGT | 110 |
|  | out reverse | CCTATCATCTCCACACTAACGCT |  |
|  | nest foward | TGTTAGGGGTGATTTTTAGAGG |  |
|  | nest reverse | ATTAACCCCATTTCCATCCTAACTATCTT |  |
|  | sequencing | TTTTAGAGGAGTTTTTTTTTTTTA |  |
| *STAP1* | out foward | AGTYATGTYTTYTGYAAATAAAAATGGAYAYY | 91 |
|  | out reverse | TTRCTTTTTAACCACCAACACTACC |  |
|  | nest foward | YYGTTTYTTTYATYTTYTGGTGATGTTAA |  |
|  | nest reverse | ARARRRCAATCTCTRRRTAATCCACATRTR |  |
|  | sequencing | GGTGATGTTAATYTTYTGTTTA |  |
