# Supplementary material for: Diagnostic and prognostic value of STAP1 and AHNAK methylation in peripheral blood immune cells for HBV-related hepatopathy
Source: Front Immunol. 2023 Jan 13;13:1091103. doi: 10.3389/fimmu.2022.1091103 (PMC9880311; doi:10.3389/fimmu.2022.1091103)
Supplement: Supplementary file 4 [file Table_2.docx]

| **Group1** | **Group2** | **P value** | **sig** |
| --- | --- | --- | --- |
| NC | CHB | 0.099220197880508 | ns |
| NC | CLC | 0.0227770456427372 | * |
| NC | DCLC | 0.0342949709616447 | * |
| NC | stage 0 HCC | 3.03991228102535e-05 | ns |
| NC | stage A HCC | 0.00318967463750472 | ** |
| NC | stage B HCC | 0.0146550902387893 | * |
| NC | stage C HCC | 0.0341840137901583 | * |
| CHB | CLC | 0.835092006082409 | ns |
| CHB | DCLC | 0.633388222057894 | ns |
| CHB | stage 0 HCC | 0.0181980371236203 | * |
| CHB | stage A HCC | 0.275183188370379 | ns |
| CHB | stage B HCC | 0.361946681721912 | ns |
| CHB | stage C HCC | 0.550640637609219 | ns |
| CLC | DCLC | 0.70755145533855 | ns |
| CLC | stage 0 HCC | 0.00370395279622849 | ** |
| CLC | stage A HCC | 0.239359586895481 | ns |
| CLC | stage B HCC | 0.372414536298935 | ns |
| CLC | stage C HCC | 0.604153638137602 | ns |
| DCLC | stage 0 HCC | 0.0685736287633304 | ns |
| DCLC | stage A HCC | 0.580897226839328 | ns |
| DCLC | stage B HCC | 0.648182107231962 | ns |
| DCLC | stage C HCC | 0.882812322819543 | ns |
| stage 0 HCC | stage A HCC | 0.135319920947867 | ns |
| stage 0 HCC | stage B HCC | 0.230355040330504 | ns |
| stage 0 HCC | stage C HCC | 0.129353841044015 | ns |
| stage A HCC | stage B HCC | 0.988869528169836 | ns |
| stage A HCC | stage C HCC | 0.726642860846739 | ns |
| stage B HCC | stage C HCC | 0.770984573062666 | ns |
