# Supplementary material for: Diagnostic and prognostic value of STAP1 and AHNAK methylation in peripheral blood immune cells for HBV-related hepatopathy
Source: Front Immunol. 2023 Jan 13;13:1091103. doi: 10.3389/fimmu.2022.1091103 (PMC9880311; doi:10.3389/fimmu.2022.1091103)
Supplement: Supplementary file 5 [file Table_3.docx]

| **Group1** | **Group2** | **P value** | **sig** |
| --- | --- | --- | --- |
| NC | CHB | 0.714720411611851 | ns |
| NC | CLC | 0.0925926936308806 | ns |
| NC | DCLC | 0.0153002700395341 | * |
| NC | stage 0 HCC | 0.00703715218785081 | ** |
| NC | stage A HCC | 0.00218634880280183 | ** |
| NC | stage B HCC | 0.0183648089111876 | * |
| NC | stage C HCC | 0.00672555857893211 | ** |
| CHB | CLC | 0.0728774519808396 | ns |
| CHB | DCLC | 0.00445867418345368 | ** |
| CHB | stage 0 HCC | 0.00111662794675505 | ** |
| CHB | stage A HCC | 0.000141471611149902 | *** |
| CHB | stage B HCC | 0.00659045604611502 | ** |
| CHB | stage C HCC | 0.00123714763698283 | ** |
| CLC | DCLC | 0.0452633747593207 | * |
| CLC | stage 0 HCC | 0.00360014519803587 | ** |
| CLC | stage A HCC | 2.70624512508973e-05 | ns |
| CLC | stage B HCC | 0.0824941151270036 | ns |
| CLC | stage C HCC | 0.00761836031558806 | ** |
| DCLC | stage 0 HCC | 0.461087084405508 | ns |
| DCLC | stage A HCC | 0.0522886493533018 | ns |
| DCLC | stage B HCC | 0.913249058734421 | ns |
| DCLC | stage C HCC | 0.434574857080957 | ns |
| stage 0 HCC | stage A HCC | 0.195514547505635 | ns |
| stage 0 HCC | stage B HCC | 0.425928859084853 | ns |
| stage 0 HCC | stage C HCC | 0.895102141360839 | ns |
| stage A HCC | stage B HCC | 0.0583694246720982 | ns |
| stage A HCC | stage C HCC | 0.324093743663737 | ns |
| stage B HCC | stage C HCC | 0.402208653009654 | ns |
