# Supplementary material for: Diagnostic and prognostic value of STAP1 and AHNAK methylation in peripheral blood immune cells for HBV-related hepatopathy
Source: Front Immunol. 2023 Jan 13;13:1091103. doi: 10.3389/fimmu.2022.1091103 (PMC9880311; doi:10.3389/fimmu.2022.1091103)
Supplement: Supplementary file 6 [file Table_4.docx]

| **Group1** | **Group2** | **P value** | **sig** |
| --- | --- | --- | --- |
| NC | CHB | 0.837907583834663 | ns |
| NC | CLC | 0.417965208424006 | ns |
| NC | DCLC | 0.357833648935452 | ns |
| NC | stage 0 HCC | 0.00117250194703217 | ** |
| NC | stage A HCC | 0.00402569641019352 | ** |
| NC | stage B HCC | 0.134990044038068 | ns |
| NC | stage C HCC | 0.366870472701104 | ns |
| CHB | CLC | 0.668125614937033 | ns |
| CHB | DCLC | 0.613274643516042 | ns |
| CHB | stage 0 HCC | 0.013665441580958 | * |
| CHB | stage A HCC | 0.0344390516972612 | * |
| CHB | stage B HCC | 0.278864043595384 | ns |
| CHB | stage C HCC | 0.569490858336937 | ns |
| CLC | DCLC | 0.925516061834167 | ns |
| CLC | stage 0 HCC | 0.0072408674154298 | ** |
| CLC | stage A HCC | 0.0244703467779782 | * |
| CLC | stage B HCC | 0.389882707360333 | ns |
| CLC | stage C HCC | 0.819401024227823 | ns |
| DCLC | stage 0 HCC | 0.00753343112801787 | ** |
| DCLC | stage A HCC | 0.0261319186195019 | * |
| DCLC | stage B HCC | 0.423823046969221 | ns |
| DCLC | stage C HCC | 0.876575660604342 | ns |
| stage 0 HCC | stage A HCC | 0.564943061771441 | ns |
| stage 0 HCC | stage B HCC | 0.161933422615394 | ns |
| stage 0 HCC | stage C HCC | 0.0402843808469878 | * |
| stage A HCC | stage B HCC | 0.329885515542348 | ns |
| stage A HCC | stage C HCC | 0.0997494631518681 | ns |
| stage B HCC | stage C HCC | 0.57395523619783 | ns |
