# Supplementary material for: Diagnostic and prognostic value of STAP1 and AHNAK methylation in peripheral blood immune cells for HBV-related hepatopathy
Source: Front Immunol. 2023 Jan 13;13:1091103. doi: 10.3389/fimmu.2022.1091103 (PMC9880311; doi:10.3389/fimmu.2022.1091103)
Supplement: Supplementary file 7 [file Table_5.docx]

| **Group1** | **Group2** | **P value** | **sig** |
| --- | --- | --- | --- |
| NC | CHB | 0.789103324369816 | ns |
| NC | CLC | 0.103245094184649 | ns |
| NC | DCLC | 0.122758603842595 | ns |
| NC | stage 0 HCC | 0.0026288429677115 | ** |
| NC | stage A HCC | 0.000291143995188175 | *** |
| NC | stage B HCC | 0.0199689700430284 | * |
| NC | stage C HCC | 0.000751835228836667 | *** |
| CHB | CLC | 0.205848200639904 | ns |
| CHB | DCLC | 0.218970130906142 | ns |
| CHB | stage 0 HCC | 0.0087248743253742 | ** |
| CHB | stage A HCC | 0.00107992929131503 | ** |
| CHB | stage B HCC | 0.0411012550283294 | * |
| CHB | stage C HCC | 0.00217233828780862 | ** |
| CLC | DCLC | 0.898126091763971 | ns |
| CLC | stage 0 HCC | 0.074190434617924 | ns |
| CLC | stage A HCC | 0.00691613731337763 | ** |
| CLC | stage B HCC | 0.229580928655981 | ns |
| CLC | stage C HCC | 0.0165331796119278 | * |
| DCLC | stage 0 HCC | 0.194031733297633 | ns |
| DCLC | stage A HCC | 0.0358691935392998 | * |
| DCLC | stage B HCC | 0.323605369451697 | ns |
| DCLC | stage C HCC | 0.0428101927363762 | * |
| stage 0 HCC | stage A HCC | 0.215929023093965 | ns |
| stage 0 HCC | stage B HCC | 0.967985762713606 | ns |
| stage 0 HCC | stage C HCC | 0.216673036378156 | ns |
| stage A HCC | stage B HCC | 0.461576067376965 | ns |
| stage A HCC | stage C HCC | 0.755279592657066 | ns |
| stage B HCC | stage C HCC | 0.378536578175308 | ns |
